# Adaptive X-ray imaging with reinforcement learning

**DOI:** 10.1107/S1600577525009592

**Published:** 2026-01-01

**Authors:** Tobias Boltz, Daniel Ratner, Samuel M. Webb

**Affiliations:** a SLAC National Laboratory, Menlo Park, CA94025, USA; Paul Scherrer Institut, Switzerland

**Keywords:** synchrotron radiation, X-ray imaging, reinforcement learning

## Abstract

X-ray imaging is a powerful technique to scan samples in a variety of contexts including biological, environmental and materials science, but commonly requires a synchrotron light source to produce X-rays at sufficient intensity. As these facilities are expensive to operate, the available beam time is limited and always in high demand. Particularly if the illuminated samples are sparse, standard raster scanning methods can be time-consuming, with a majority of that time being spent on areas of the image that carry little information. To increase the efficiency and maximize the information gain for a given time budget, we split the scanning process into a series of steps where previous measurements are used to inform the decision making and adapt the exposure distribution at later stages of the sequence. We formulate this task as a reinforcement learning problem where the goal is to produce a sequence of exposure maps that maximize a predefined scalar metric. We demonstrate the potential of this approach in simulations where the adaptive illumination can accelerate the measurement process by up to an order of magnitude compared with standard raster scanning. Finally, we present the first results from deploying the trained agents on an X-ray fluorescence beamline at the Stanford Synchrotron Radiation Lightsource.

## Abstract 2

We present a reinforcement learning based approach to X-ray imaging which allows for up to an order of magnitude lower exposure time to reach the same image quality as with a standard raster scan. This is achieved by splitting the imaging task into several steps and letting initial measurements inform the exposure distribution at later stages of the sequence.

## 1. Introduction

Synchrotron-radiation-based X-ray analysis methods are often utilized for the location and characterization of materials because X-rays are highly penetrating, are generally non-destructive, and provide unique and rich chemical and microstructural information. Standard X-ray imaging applications typically rely on a series of raster scans, where each pixel of the ground-truth sample is illuminated by a probe beam over a constant predefined exposure time. While this simplifies technical implementations, allows for straight­forward reconstruction techniques and keeps individual measurements easy to interpret, it commonly is not a very efficient approach from an information-theoretical perspective. Particularly in cases where signals are sparse, the majority of pixels will carry little information but are illuminated at the same rate as the regions of interest. Understanding and improving the efficiency of raster scanning is thus a high-priority broadly applicable research target. If the distribution of exposure time was instead tailored to the specific structure of the image, the same reconstruction quality could be achieved with a much lower overall illumination time. Or, alternatively, the regions of interest could be reconstructed with significantly higher fidelity using the same overall time budget. As an additional benefit, a more efficient distribution of the illumination time can also reduce sample damage caused by the incident X-ray beam. We thus split the data acquisition into several steps where initial measurements may inform the exposure distribution at later stages in the sequence. This allows for an adaptive approach that progressively adjusts to the individual sample as information about the underlying structure is acquired. In principle, this can be achieved by manually adjusting the region of interest and tuning the corresponding exposure times in between steps. However, as this requires additional time for analysis and comes with major feasibility constraints regarding granularity and precision, we resort to a machine learning (ML)-based approach.

Our method is related to previous work on ML-based data acquisition strategies in various domains, including medical imaging tasks like magnetic resonance imaging (MRI) (Zhang *et al.*, 2019 ▸) and computed tomography (CT) scans (Shen *et al.*, 2021 ▸). Optimizing the data acquisition process at synchrotron light sources is of particular interest due to continually increasing data rates and high operating costs. In contexts with weak priors, previous work has explored the application of Bayesian optimization (Fong *et al.*, 2021 ▸) and related methods including kriging (Noack *et al.*, 2019 ▸), Bayesian algorithmic execution (Chitturi *et al.*, 2024 ▸) and Bayesian optimal experimental design (Chen *et al.*, 2023 ▸). Alternatively, re­inforcement learning (RL) may enable various advantages. For example, when strong priors exist, policy-based approaches can exploit these priors to achieve greater efficiency. Moreover, a deep-learning policy can be fast (*e.g.* when decisions are needed with sub-second latency) and can support high-dimensional inputs and outputs. RL has also been used previously on light sources, including by Maffettone *et al.* (2021 ▸) who demonstrated RL acquisition on the National Synchrotron Light Source II (NSLS-II) beamlines, and Kandel *et al.* (2023 ▸) who used a supervised learning approach to guide data acquisition in X-ray microscopy scans at the Argonne Photon Source (APS). Here, we focus specifically on training an RL agent to optimize the selection of exposure time in XRF imaging.

Expanding on previous work (Betterton *et al.*, 2020 ▸), we formalize the task as an RL problem and explore solution methods based on convolutional neural networks (CNNs). To demonstrate the potential of this approach, we model the measurement process in offline simulations using randomly generated ground-truth images and simple Poisson statistics. By approximating the Poisson noise with a Gaussian distribution we obtain a fully differentiable simulation and can thus train RL agents with direct policy gradients. We then evaluate the trained agents on a selection of real-world samples to validate their performance on realistic, previously unseen, images. Finally, we discuss our first results from deploying agents on an X-ray fluorescence (XRF) beamline at the Stanford Synchrotron Radiation Lightsource (SSRL).

This advance in data collection is applicable to many areas of science that use XRF imaging, for instance locating particles for nuclear forensics (Edwards *et al.*, 2022 ▸; van Veelen *et al.*, 2022 ▸), location of microparticles of rare earth elements in fly ash (Rivera *et al.*, 2017 ▸; Liu *et al.*, 2019 ▸; Stuckman *et al.*, 2018 ▸; Hedin *et al.*, 2019 ▸) and characterization of small deposits of critical materials in geologic ores (Ryan *et al.*, 2013 ▸). This adaptive scanning methodology can be combined with advanced synchrotron-based X-ray techniques (*e.g.* X-ray absorption spectroscopy, high-energy-resolution fluorescence detection X-ray absorption spectroscopy or X-ray diffraction) to enhance extraction of signatures and characteristics from located particles.

## 2. Problem definition

While generally following the theoretical description given by Betterton*et al.* (2020 ▸), we introduce a few simplifications and small modifications to facilitate an initial experimental demonstration. The general task we are concerned with is the illumination of a ground-truth sample with an X-ray beam, measuring its response pixel by pixel. For simplicity, here we assume a constant X-ray beam intensity and that the desired beam size can be achieved through focusing rather than the use of apertures. By dividing the process into several steps, we facilitate an information-driven adaptive approach where the illumination can be tailored towards the specific structure of the sample.

### 2.1. Sequential imaging

Consider a ground-truth image 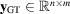 and a total illumination budget of *nm*τ_total_, where τ_total_ denotes the average dwell time per pixel. The total budget is distributed over *T* steps, where at each step *t* ∈ {1,…, *T*} we illuminate the sample according to an exposure map 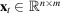 and observe a corresponding intensity reading 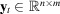. The exposure maps define the spatial distribution of illumination time at each step within the constraints 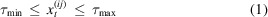
and 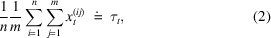
where the average dwell times τ_*t*_ are determined beforehand and satisfy 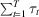 = τ_total_. Given a sequence of exposure maps (**x**_1_,…, **x**_*T*_) and corresponding measurements (**y**_1_,…, **y**_*T*_), we then generate a prediction 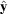 of the ground-truth image by computing the weighted sum 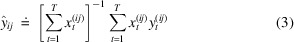
with the goal of minimizing the mean-squared error 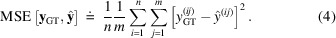
In this work, we are primarily concerned with the task of choosing, at each step, that exposure map which leads to the minimum final prediction error.

### 2.2. Reinforcement learning

As a computational approach to sequential decision problems, RL is ideally suited to formalizing the sequential imaging task defined above. Instead of learning from a pre-existing data set, RL is concerned with learning from interaction with an environment. The learner or decision maker, usually called the agent, iteratively interacts with the environment while attempting to improve its behavior based on the general concept of trial-and-error search. At each iteration, the agent observes the current state of the environment *S*_*t*_ and is faced with the decision to choose an action *A*_*t*_. The agent’s behavior is thus represented by a policy 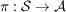 that maps the perceived state to an action. As a response to the chosen action, the environment yields a scalar reward *R*_*t*_ and transitions to the next state *S*_*t*+1_. Thereby, the agent’s overarching goal is defined as being to maximize the cumulative reward received over time, *i.e.* the discounted return, 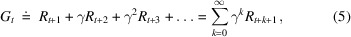
where γ denotes a discount factor 0 ≤ γ ≤ 1 to ensure convergence. A more detailed introduction to the field of RL can be found in the work of Sutton & Barto (2018 ▸).

We formalize the sequential imaging task as an RL problem by defining the corresponding state, action and reward. In order to provide the agent with sufficient information to make decisions, the state is defined as 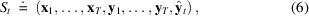
where not-yet-obtained exposure maps and measurements at time *t*, *i.e.* {**x**_*i*_, **y**_*i*_ with *i* > *t*}, are filled in with zero matrices. Based on the given state, the agent’s policy is queried to choose an action from the set 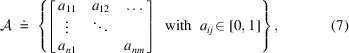
where any chosen action *A*_*t*_ is mapped to a physically valid exposure map **x**_*t*_, satisfying equation (1), through a linear rescaling function. In order to define a reward that expresses the notion of focusing on the most relevant parts of potentially sparse images, we introduce the subset 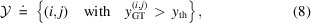
which contains only pixels above a given signal threshold *y*_th_. We then formalize the reward as 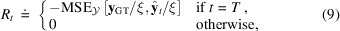
with the normalization factor ξ. The agent’s performance is thus exclusively measured by the final prediction error on the subset of pixels that are considered signal.

### 2.3. Gaussian noise approximation

In this work, we assume that the intensity readings **y** measured in response to an exposure map **x** follow a Poisson distribution given by 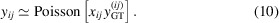
We introduce the Gaussian approximation 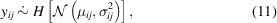
with mean and variance given by 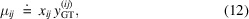

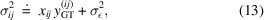
the minimum noise level 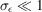 and the Heaviside function 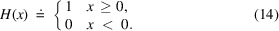
By using this approximation, we obtain an environment where, outside of encountering *H*(0), measurements **y**_*t*_ are fully differentiable with respect to the corresponding exposure map **x**_*t*_. More generally, this means that all state transitions from *S*_*t*_ to *S*_*t*+1_ are differentiable with respect to the chosen action. This is an important simplification of the general RL problem as it allows training the agent with direct policy gradients, *i.e.* we can directly calculate the gradient of the expected return, 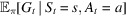, with respect to the policy π and its parameters. As the Heaviside function in equation (11) breaks this differentiability, we use a straight-through estimator during back propagation, *i.e.* we replace the Heaviside function with the identity function when computing gradients.

## 3. Simulation

In order to explore the potential of the adaptive approach outlined above, we define a simulation environment based on equation (11) and a simple generator to produce randomized ground-truth images. The signal in these 200 × 200 pixel images is limited to one to five rectangular patches with a length of two to seven pixels in each dimension. Individual pixel values are then sampled from a uniform distribution while guaranteeing values above the signal threshold *y*_th_ = 1000 and maintaining a fixed signal-to-background ratio of*S*/*B* ≃ 143. Given these ground-truth images **y**_GT_, we perform scans over a total of three steps with average exposure times of 5 ms, 5 ms and 15 ms, and the limitations τ_min_ = 3 ms and τ_max_ = 998 ms. As there is no information about the ground-truth image before the initial measurement **y**_1_, we always use a uniform first exposure map, *i.e.*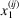 = τ_1_. The agent’s entire task is thus to choose the second and final exposure map that optimizes the objective defined in equation (9), with the normalization factor ξ set to the average signal value in the prediction target **y**_GT_. To be able to process arbitrary image shapes, we represent the agent’s policy π as a fully convolutional neural network that processes the states defined in equation (6) and returns an action from the set defined in equation (7).

**Figure 1:**
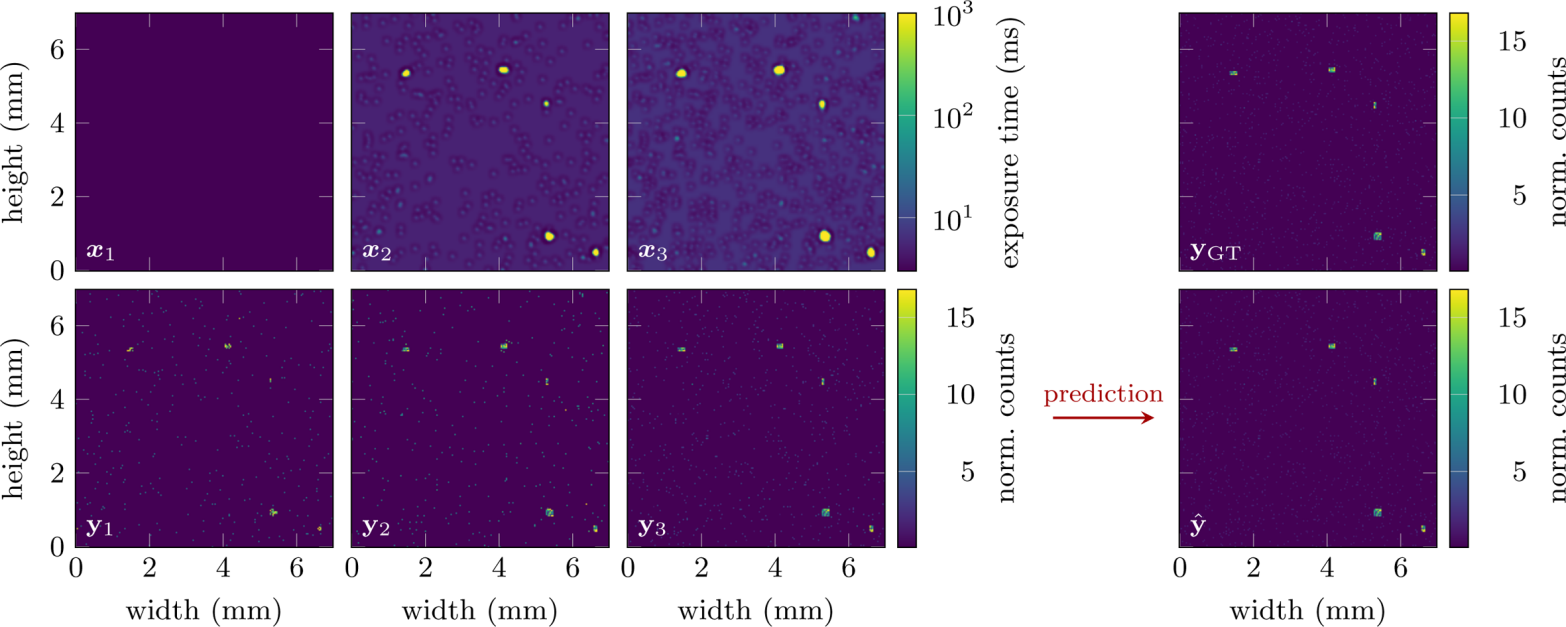
Example episode illustrating the agent’s adaptive policy after training. The information acquired through measurements **y**_1_ and **y**_2_ is used to emphasize exposure on the five patches of signal when generating exposure maps **x**_2_ and **x**_3_. By computing the weighted sum defined in equation (3), we obtain the final prediction of the ground-truth image.

**Figure 2:**
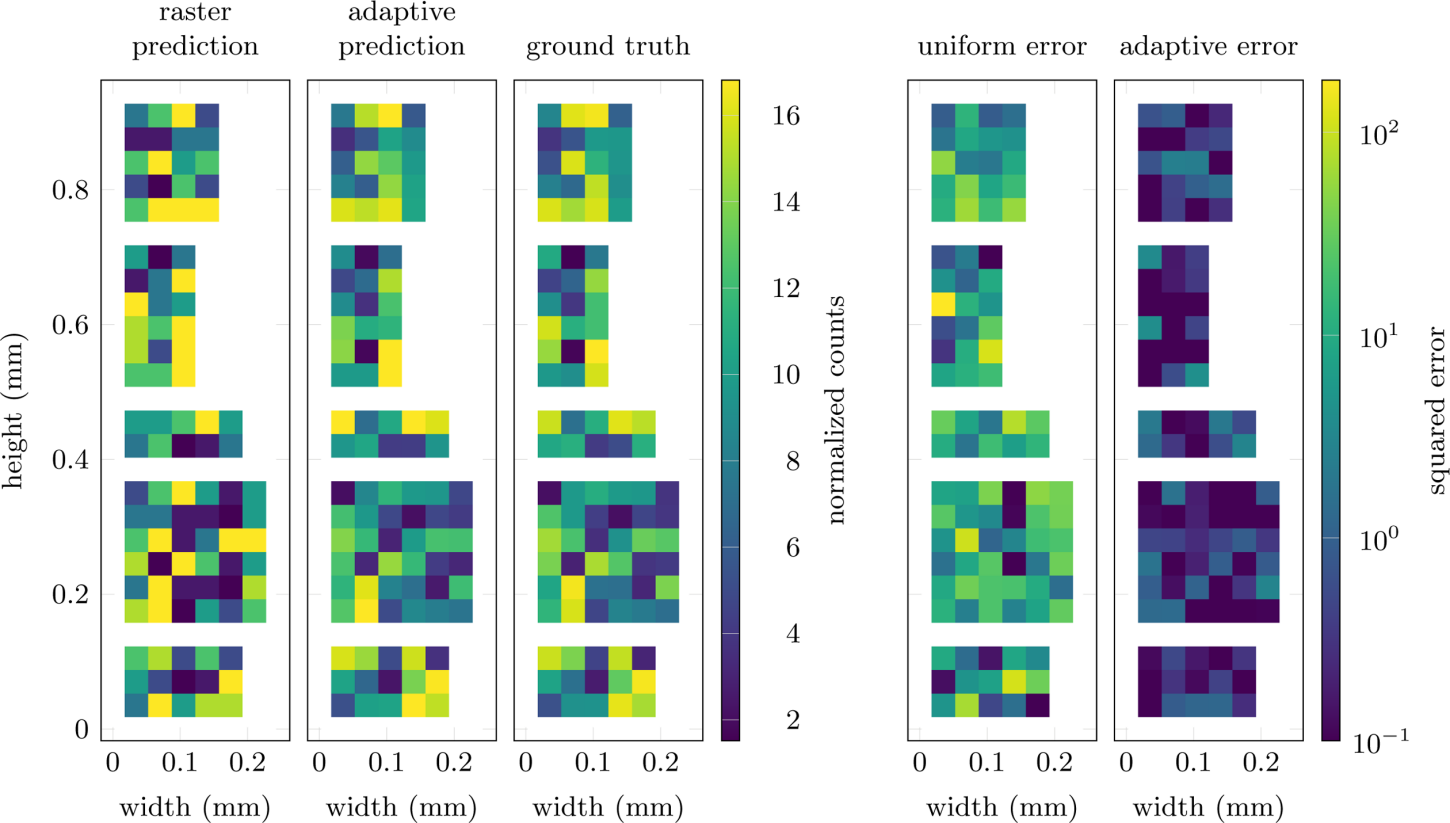
Prediction of randomly generated signal patches in simulations. Compared with the results from a raster scan (first column), the adaptive approach (second column) more accurately predicts the five signal patches in the ground-truth image (third column). This leads to up to an order of magnitude lower prediction error, illustrated by the pixel-wise squared errors on the right (fourth and fifth columns).

We keep the network relatively small at a total of three hidden layers with 14 channels, a kernel size of 3–7 and rectified linear unit (ReLU) activation functions. This mainly serves the purpose of avoiding overfitting on the artificial training data set, but also reduces the computational cost and speeds up training. Finally, the output layer features a sigmoid activation function to ensure all outputs are within [0, 1]. The agent is then trained over a total of 20000 episodes, with policy updates being computed each time a batch of ten episodes has been collected. We choose to update the policy this way, rather than at each individual step, to reduce variance and because non-zero rewards occur only at the end of each episode. The adaptive behavior of the trained agent is shown in Fig. 1 ▸. After the initial measurement **y**_1_, the agent progressively adjusts to the structure of the sample and focuses on the most relevant areas of the image. This allows for the high accuracy of the final prediction illustrated in Fig. 2 ▸. Compared with the prediction obtained from a standard raster scan, the agent achieves a significantly lower residual error. This corresponds to a final reward of *R*_3_ = −6.35 × 10^−3^, compared with *R*_3_ = −1.92 × 10^−1^ for the raster scan.

**Figure 3:**
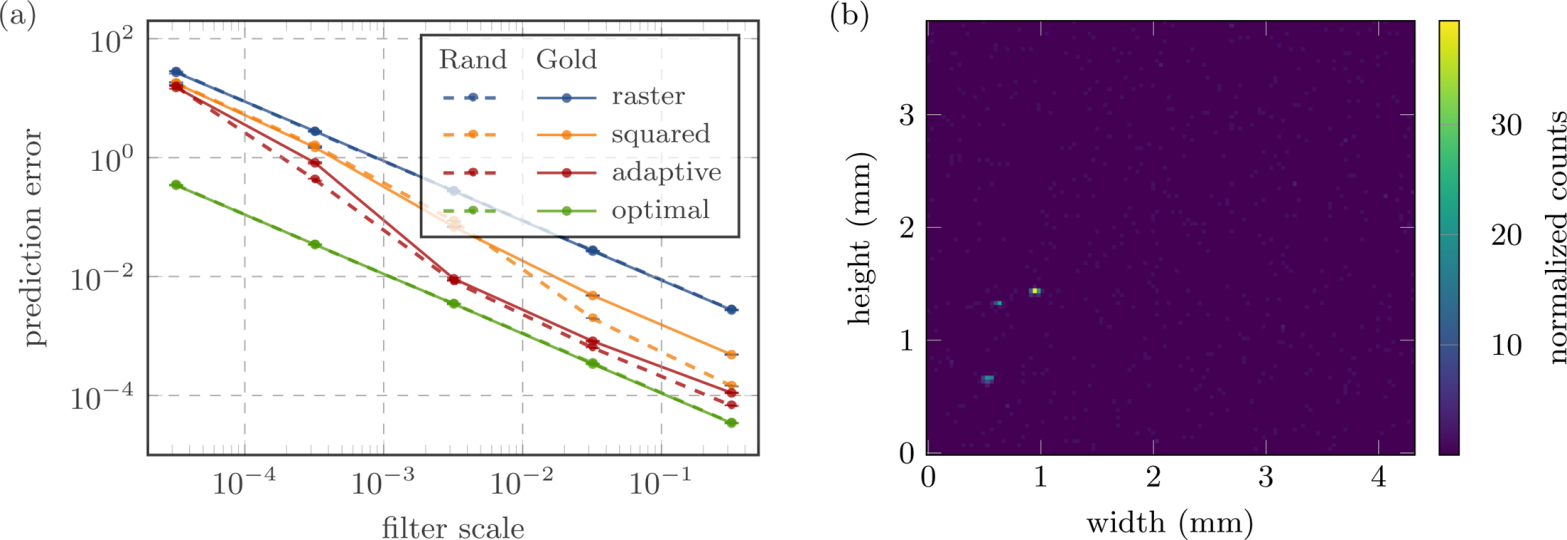
(*a*) Prediction errors in the simulation environment using both randomly generated samples (dashed lines) and patterns taken from an actual measurement of gold particles (solid lines) across different filter levels. With increased filter scaling, the adaptive policy (red) starts to outperform significantly both the raster scan (blue) and a simple baseline which uses the squared previous measurement as the exposure distribution (orange). At high filter levels, the adaptive policy eventually approaches the theoretical limit (green). Each point shows the average prediction error over 1000 episodes. (*b*) Measurement of gold particles on the XRF beamline at SSRL, which was used to evaluate the trained agent under more realistic conditions.

As this improved prediction quality is dependent on the information provided by the initial measurements **y**_1_ and **y**_2_, the benefits of the adaptive approach scale with the signal intensity in these measurements. Consider, for example, the extreme case of a signal-to-background ratio of *S*/*B* = 1.0. In that case, the agent has no information to work with and thus cannot outperform a standard raster scan. On the other hand, with very high signal-to-background ratios, a standard raster scan might already provide a prediction with satisfactory accuracy. In order to understand this dependency better, we introduce an additional filter scale factor *s*_F_ which reduces the signal observed in response to a given exposure map, that is, we modify equation (10) to 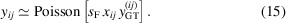
Depending on the value of *s*_F_, we thus obtain measurements with varying levels of accuracy, even if the exposure times *x*_*ij*_ are fixed. Fig. 3 ▸(*a*) illustrates how this impacts the agent’s performance (red line) compared with the standard raster scan approach (blue line). We also compare the agent’s performance against a simple baseline which uses the squared previous measurement as the exposure distribution (orange line) and the upper limit of an optimal policy which receives the signal location *a priori* (green line).

While the agent offers limited benefits at very low filter scaling, *s*_F_ < 1 × 10^−4^, its performance continually improves with access to more information and approaches the theoretical limit at high filter values. While the agent’s average prediction error is only a factor of 1.8 lower than that from a raster scan at *s*_F_ = 3.2 × 10^−5^, it reaches factors of 33.5 and 43.2 at *s*_F_ = 3.2 × 10^−3^ and *s*_F_ = 3.2 × 10^−2^, respectively. Employing an adaptive approach could thus improve the accuracy of the final image by more than an order of magnitude. Alternatively, reaching a comparable image quality with a raster scan would require at least an order of magnitude longer exposure times. In fact, compared with the prediction error achieved through the adaptive approach at *s*_F_ = 3.2 × 10^−3^, almost two orders of magnitude longer exposure times are required to yield comparable results.

While these simulation results are promising, there is no guarantee that the trained policies generalize to more realistic images. We thus evaluated the same agents, trained on randomly generated data, on a measurement of gold particles taken on the XRF beamline at SSRL, shown in Fig. 3 ▸(*b*). The results are displayed as solid lines in Fig. 3 ▸(*a*), verifying that the trained agents can also be deployed under more realistic conditions. Although the agent’s performance is slightly worse than on purely simulated data, it still reaches factors of 29.8 and 32.7 lower prediction errors when compared with a raster scan at *s*_F_ = 3.2 × 10^−3^ and *s*_F_ = 3.2 × 10^−2^, respectively.

## 4. Experimental setup

XRF images were collected at the Stanford Synchrotron Radiation Lightsource (SSRL) using beamline 7-2. The incident X-ray energy was obtained using an Si(111) double-crystal monochromator tuned to 13.45 keV with the Stanford Positron Electron Asymmetric Ring (SPEAR3) storage ring containing 500 mA at 3.0 GeV in top-off mode. The fluorescence lines of the elements of interest were monitored using a seven-channel silicon drift Vortex detector (Hitachi) using Xspress3 pulse processing electronics (Quantum Detectors). The beam was focused to 35 µm using a polycapillary (XOS Optics). The nominal optimized flux of the beamline at this energy was approximately 10^11^ photons per second. Filtering of the beam was achieved through in-line metal foil attenuators (Al or Cu) which reduced the flux to present a maximally challenging measurement scenario. The filters reached attenuation factors of 30× to 160×, resulting in fluorescence count rates from the sample of 4 to 600 counts per 5 ms and pixel.

Samples were mounted at 45° to the incident X-ray beam and used a Newport IMS1000 for horizontal motion and IMS300V for vertical motion, with a Newport XPS motor driver for motion control. The samples were fabricated by depositing 50 µm gold particles on Nucleopore polycarbonate filters with a 0.2 µm pore size. All generated exposure maps are post-processed by a software check to ensure compatibility with the physical limitations of the Newport motors. If differences in dwell times between neighboring pixels are found to be incompatible, simple linear ramps are added to accelerate or decelerate on preceding pixels such that the targeted dwell times can be met. Thereby, the pixels with the highest exposure time are prioritized and the overall illumination time of the sample is re-normalized to ensure comparability with different runs. The post-processed exposure maps are subsequently passed to the Newport XPS motion controller software which generates unidirectional line-by-line trajectories which target the overall dwell time requested in each pixel in the exposure map. These trajectories are utilized by the motion controller to adjust the speed of the stage motors during each trajectory line. While we kept all trajectory positions fixed, with only the exposure time varying, a potential future improvement would also include more complex trajectory planning, such as to visit only the points of interest. The Newport XPS motor driver uses the encoded position of the motors to send trigger pulses each time a defined pixel has been traversed during the trajectory. These pulses are used to gate the Xspress3 detector electronics and advance data collection to the next pixel.

As the used CNNs are relatively small, *i.e.* three hidden layers with 14 channels and a kernel size of 3, the computation time needed for their inference and the generation of the exposure maps is negligible compared with the overall scanning time. For example, the average computation time for a single exposure map is around 79 ms on an Intel Core i5-7200U at 2.50 GHz with two physical cores. To estimate the full scanning overhead, we compare the total data collection time of a nominal raster scan against that of the trained agent. While the former uses uniformly distributed dwell times of 25 ms per pixel, the latter adjusts the distribution and deploys the same overall exposure time adaptively. This yields a data collection average of 1903 ± 10 s across 29 runs for the raster scans and 1933 ± 16 s across 25 runs for the agent on one of the selected samples. The overheads due to agent calculations and adaptively changing acceleration and deceleration are thus of the order of 1.5% of the total scanning time.

Efforts to deploy the agents trained on simulations on beamline 7-2 come with several practical challenges.

Firstly, the achievable exposure time at each individual pixel is limited by the physical setup, *i.e.* the motor which moves the sample horizontally, relative to the X-ray beam, in order to achieve the desired dwell times. This limits the difference in exposure times between neighboring pixels due to the finite maximum acceleration achievable by the motor. We use this physics check to validate, and if necessary correct, planned exposure maps in terms of their practical feasibility. The effective exposure times can thus deviate from the exact values generated by the agent.

Secondly, the spatial precision of the motor movements may result in discrepancies between the planned and true exposures and between the true and acquired images, impacting the comparison of measured images on a pixel by pixel basis. As we conduct our measurements at a pixel size of 35 µm, small disturbances can easily translate to a spatial mismatch of up to a full pixel. While we ideally aim for a loss-less translation from the original planned exposure to the actual trajectories executed by the motor, small inaccuracies in hardware and software can quickly lead to inaccurate exposure maps and result in high prediction errors, due to the resulting spatial mismatch between the predicted image and the ground truth.

Thirdly, the simulation assumes a pure Poisson distribution, where the expected value for each pixel is exclusively determined by the ground-truth image and the exposure time. This neglects noise contributions from a variety of other sources, such as from electrical components, detector inaccuracies or disturbances due to scattering effects on the sample. A thorough analysis of these different contributions remains a subject for future work.

## 5. Experimental demonstration

**Figure 4:**
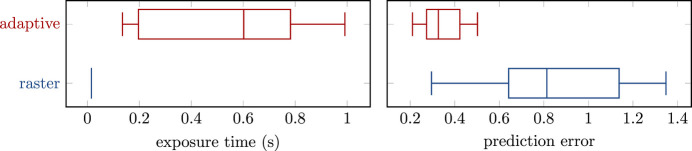
(Left) Average exposure times and (right) average prediction errors for raster scans (blue) and adaptive scans (red) on the XRF beamline at SSRL. Shown are the distributions over the average values of τ and 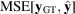 within the subset of pixels defined as signal 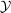 and across a total of nine runs each. The increased exposure times in the adaptive scans indicate that the agent has correctly identified and emphasized the signal region while maintaining the same overall scanning time.

**Figure 5:**
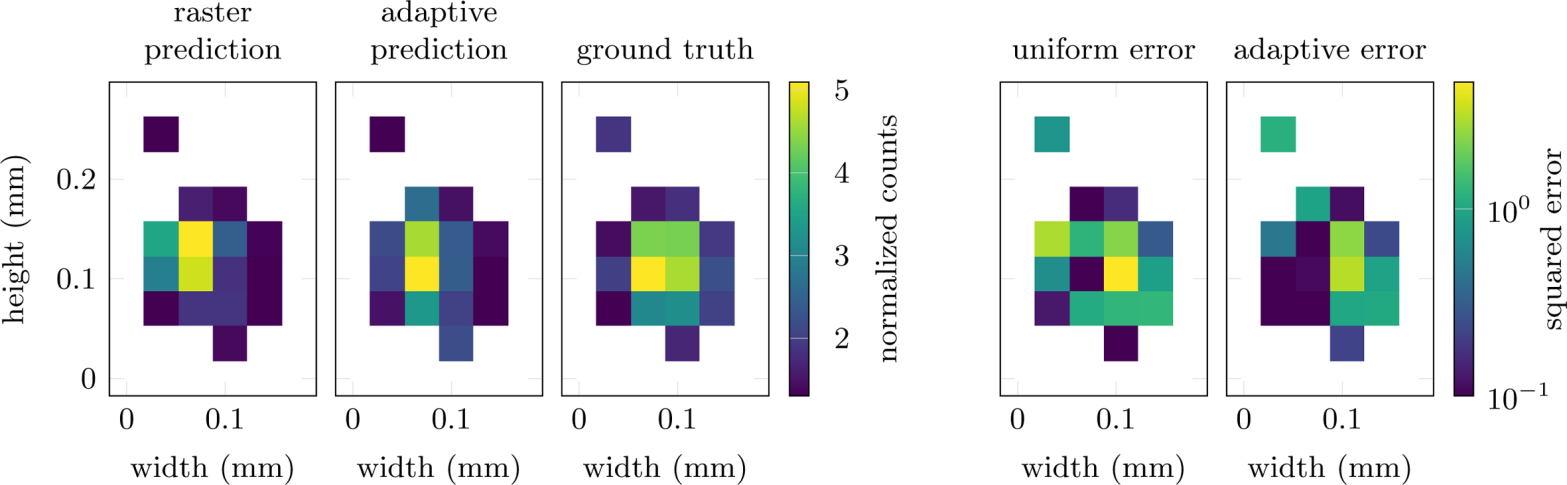
Average predictions of the signal patches in measurements on the XRF beamline at SSRL. Compared with the results from the raster scans (first column), the adaptive approach (second column) more accurately predicts the signal patches in the ground-truth image (third column) due to the increased exposure time. This corresponds to a factor of two lower prediction error, illustrated by the pixel-wise squared errors on the right (fourth and fifth columns).

In order to evaluate the trained agents in measurements on the XRF beamline at SSRL, we initially measured a range of 100 × 100 pixel samples at τ = 200 ms dwell time which serve as the ground-truth images, *i.e.* the target for all predictions. We then performed fast scans with average dwell times of 5 ms, 5 ms and 15 ms at a filter level of *s*_F_ = 1.56 × 10^−2^ with uniform and adaptive exposure maps, respectively. Measurements of the signal-to-background ratio under these conditions yielded a value of *S*/*B* = 10.55, which makes the identification of signal areas a major challenge. We typically observed between 0 and 3 counts per pixel in the initial scans at τ_1_ = 5 ms. The trained agent responds to these conditions by prioritizing areas where a few neighboring pixels yield non-zero count levels. This eventually leads to a 40 times higher exposure on the subset of signal pixels 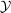, as shown in Fig. 4 ▸. Compared with a median exposure time of τ = τ_3_ = 15 ms, the adaptive approach yields a median exposure of τ = 602 ms and occasionally even reaches the maximum allowed dwell time of 998 ms per pixel. Although we observed a significant amount of variance from run to run, the adaptive approach consistently yields at least an order of magnitude longer exposure time on the signal area. This demonstrates the agent’s capability of identifying the areas of interest under practical conditions. The increased exposure generally translates to a lower prediction error, as shown on the right hand side of Fig. 4 ▸. With a median prediction error of 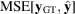 = 0.33, the error is roughly a factor of 2.5 lower than that for the raster scans with a median of 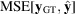 = 0.81. This is further illustrated by the average predictions in Fig. 5 ▸, where the adaptive approach clearly yields a more accurate prediction of the ground truth.

Overall, these results demonstrate the agent’s capability of adapting to the structure of the sample and allocating exposure time accordingly. The extended exposure time translates to a higher accuracy in predicting the signal areas in the ground-truth images. While the simulation results shown in Fig. 3 ▸ yield improvements of up to an order of magnitude, the difference is not as pronounced in the measured prediction errors. This is probably a consequence of the limited temporal and spatial precision in executing the planned exposure maps and measuring the corresponding response. For example, the squared errors in Fig. 5 ▸ show that the left half of the main signal patch is predicted with higher accuracy than the right half. This might be the result of additional effects like scattering or detector noise, as discussed in Section 4, and is a subject for future studies.

## 6. Conclusion and outlook

In this work we have demonstrated experimentally how X-ray imaging can be carried out more efficiently by splitting the scanning process into several steps and adaptively distributing exposure time. With a fixed total exposure time, the adaptive distribution allows higher dwell times in the most relevant areas and thus reduces the prediction error, *i.e.* it increases the image quality. Alternatively, the same approach can be used to accelerate the measurement process while yielding the same image quality. We show in simulations that the adaptive approach is most effective when the initial exposure is high enough to provide meaningful information about the structure of the image, yet the overall time budget is not sufficient to yield satisfactory image quality with a standard raster scan approach. While the specific gains will depend on the sample sparsity and other factors, in these examples we observe almost two orders of magnitude improvement in exposure efficiency in simulations and a factor of ten in experiments.

In practice, the benefits of this method fundamentally depend on the capabilities of the physical measurement setup. Limits in temporal and spatial precision will affect the accuracy of the generated exposure distribution and the final image quality. The minimum and maximum dwell times, as well as the maximum acceleration, all represent boundary conditions for the adaptive distribution of exposure time and thereby limit the agent’s performance.

Future improvements on the beamline will include updated detector electronics which will allow shorter effective dwell times, decreasing the minimum exposure time by at least an order of magnitude. The current sample-stage setup is designed to be able to accommodate large sample areas and requires a substantial cantilevered load. Acceleration limitations on the motion control may be improved by changing the weight distribution of the sample-stage design specifically for these applications to have more of the sample mass centered over the stage.

As the applicability of the presented method is mainly contingent on the underlying data distribution, *i.e.* the reduction of noise at higher exposure times, it should also be transferable to other imaging tasks outside of XRF scanning. While we assume a pure Poisson distribution and employ a simple statistical reconstruction method here, an alternative and more generalizable approach is to use a separate neural network for the reconstruction task. Simultaneous training of the policy and the reconstruction network comes with its own challenges, but adds further flexibility to adapt to alternative data distributions. As an additional benefit, the adaptive distribution of exposure time can also be used to reduce sample damage by decreasing the overall illumination time or by explicitly introducing an additional, specifically tailored, term in the reward function.

The method is also not necessarily limited to regression problems. In this work, we focus on minimizing the mean-squared error in the signal region, yet a similar approach can be taken for a classification task where *e.g.* the final objective is the identification of gold particles.

Finally, if the physical measurement setup is capable of supporting more complicated trajectories, the action space can also be adjusted accordingly. In this first demonstration, we restricted the agent’s action space to complete exposure maps with a fixed time budget for each step in the sequence. Allowing the agent to distribute exposure time freely between different steps, as well as to skip selected rows of the image completely, would yield additional time savings or even better image quality. Ultimately, rather than predicting exposure maps, an agent with full trajectory control would deliver the best performance. We also note that the current exposure predictions could be integrated with human-in-the-loop control, *e.g.* suggesting regions for manual follow-up, or allowing manual selection of regions for the agent to exclude or examine more closely on subsequent scans. We believe these are promising directions for future work.

## Data Availability

The data sets generated during the current study are available from the corresponding author on reasonable request.
